# Influence of Honeybee Sting on Peptidome Profile in Human Serum

**DOI:** 10.3390/toxins7051808

**Published:** 2015-05-22

**Authors:** Jan Matysiak, Agata Światły, Joanna Hajduk, Joanna Matysiak, Zenon J. Kokot

**Affiliations:** 1 Department of Inorganic & Analytical Chemistry, Poznan University of Medical Sciences; 6 Grunwaldzka Street, Poznań 60-780, Poland; E-Mails: agata_swiatly@wp.pl (A.S.); jo.hajduk@gmail.com (J.H.); zkokot@ump.edu.pl (Z.J.K.); 2 Ward of Paediatric Diseases, L. Perzyna Regional Unified Hospital in Kalisz 79 Poznańska Street, Kalisz 62-800, Poland; E-Mail: jkamatysiak@gmail.com

**Keywords:** honeybee venom, peptidome profiling, MALDI, sting

## Abstract

The aim of this study was to explore the serum peptide profiles from honeybee stung and non-stung individuals. Two groups of serum samples obtained from 27 beekeepers were included in our study. The first group of samples was collected within 3 h after a bee sting (stung beekeepers), and the samples were collected from the same person a second time after at least six weeks after the last bee sting (non-stung beekeepers). Peptide profile spectra were determined using MALDI-TOF mass spectrometry combined with Omix, ZipTips and magnetic beads based on weak-cation exchange (MB-WCX) enrichment strategies in the mass range of 1–10 kDa. The samples were classified, and discriminative models were established by using the quick classifier, genetic algorithm and supervised neural network algorithms. All of the statistical algorithms used in this study allow distinguishing analyzed groups with high statistical significance, which confirms the influence of honeybee sting on the serum peptidome profile. The results of this study may broaden the understanding of the human organism’s response to honeybee venom. Due to the fact that our pilot study was carried out on relatively small datasets, it is necessary to conduct further proteomic research of the response to honeybee sting on a larger group of samples.

## 1. Introduction

The diagnostic algorithm for *Hymenoptera* venom hypersensitivity (insect sting allergy) is a serious issue in allergological practice. Measurement of specific IgE-antibodies’ (sIgE) concentration and skin tests represent the routinely used methods to demonstrate the response of the organism to the honeybee sting [1,2]. However, these tests are not sufficient for a proper diagnosis, because of their non-specificity. A number of prospective studies analyzing the biochemistry response of the organism to the venom have been published in the last decade [1,2,3,4,5,6]. Additionally, the reaction after a bee sting at the metabolomic level was studied by our group [7]. However, there is still a lack of work analyzing the human organism’s response to *Hymenoptera* sting at the proteomic or peptidomic level.

Proteomics is one of the most promising approaches for the identification of potential biomarkers and for assessing differences between individuals of different health status. Searching for characteristic indicators of physiological or pathophysiological state related to allergic diseases, like asthma or chronic obstructive pulmonary disease, can lead to the implementation of specific and accurate diagnostic methods, noninvasive monitoring of the condition and new drug development [8,9]. The clinical research employed two proteomics strategies in the field of biomarker searching: the classical approach and peptide profiling. In the classical approach, proteins/peptides are first separated by applying two-dimensional electrophoresis (2DE), then isolated and identified using the MS/MS technique [10], which is both sample and time consuming and becomes very tedious, particularly when the analyses are repeated several times. The major aspect of the second approach is not the identification of particular proteins/peptides, but the development of peptide-protein patterns of a whole sample, called proteome pattern analysis or proteome profiling. The goal of this methodology is the recognition of changes in the proteome/peptidome, which distinguish the studied groups [11]. The description of correlations within the panels of multiple peptides representing markers of a given morbid unit provides grounds for designing rapid, specific and sensitive diagnostic tests. The multi-component peptide profiling manifests higher specificity than individual un-correlated markers do [11]. There are many studies using this approach for the diagnosis of cancer [12,13,14] and other diseases, like endometriosis, adenomyosis [15] and acute hepatitis E [16]. MALDI-TOF MS (matrix-assisted laser desorption/ionization time-of-flight mass spectrometry) is widely used for searching biomarkers in biological samples [12,16], because of its high sensitivity, high speed of analysis, low consumption of analyte and the prevalence of singly-charged ions [17]. However, the complexity of serum samples makes proteomic characterization difficult. This is caused by the presence of high abundant proteins and peptides, which might mask low abundant components with predictive, prognostic or diagnostic potential [18]. This limitation on the dynamic range may be partially overcome by using depletion methods that yield a defined subset of the proteome [19,20]. The aim of this study was to explore the serum peptide profiles from honeybee stung and non-stung individuals. The advanced chemometric analysis was applied to evaluate the organism response to honeybee sting at the peptidomic level. Since the discriminating pattern of dozens of peptides formed by a subset of *m*/*z* (mass-to-charge ratio) signals has been taken into account, no identification of single compounds was required. We decided to use three serum sample preparation procedures, including different enrichment strategies: magnetic beads based on weak-cation exchange (MB-WCX) and two types of micropipette tips with prepacked C18 reverse phase (Omix and ZipTips). Using this techniques allows the preconcentration and purification of serum samples and led to generating complementary peptide profiles. This is the first report in the available literature on clinical studies that is focused on the characterization of serum peptidomic profiles after a sting.

## 2. Results and Discussion

**Figure 1:**
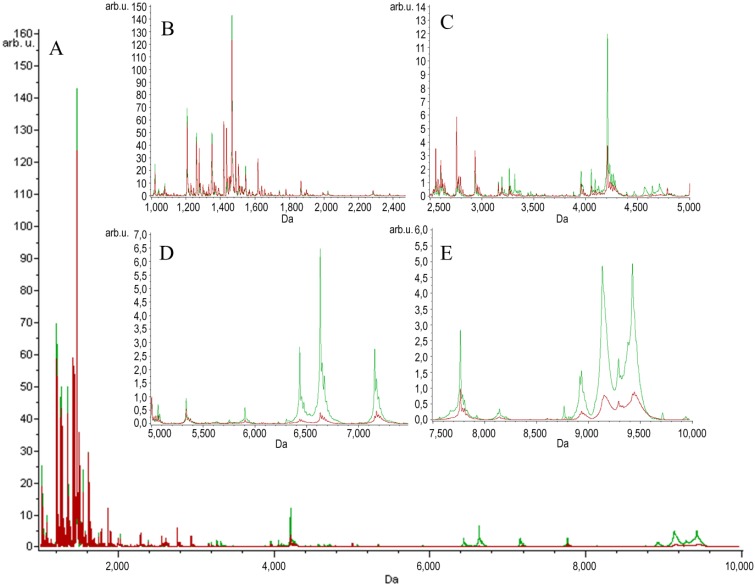
Average MALDI-TOF spectra of serum samples of stung (red) and non-stung (green) individuals pretreated with Omix over the full scan range of *m*/*z* 1–10 kDa (**A**); zoomed spectra over the *m*/*z* 1–2.5 kDa range (**B**); zoomed spectra over the *m*/*z* 2.5–5.0 kDa range (**C**); zoomed spectra over the *m*/*z* 5–7.5 kDa range (**D**); zoomed spectra over the *m*/*z* 7.5–10 kDa range (**E**).

**Figure 2:**
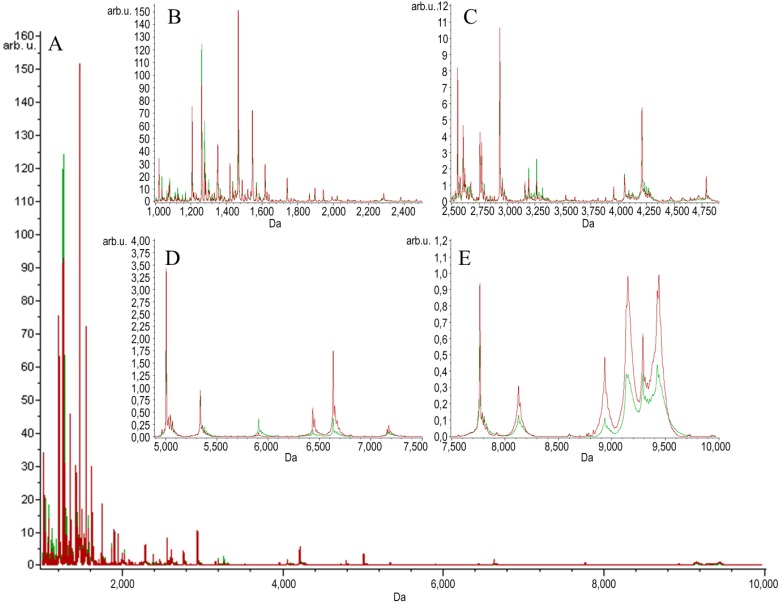
Average MALDI-TOF spectra of serum samples of stung (red) and non-stung (green) individuals pretreated with ZipTips over the full scan range of *m*/*z* 1–10 kDa (**A**); zoomed spectra over the *m*/*z* 1–2.5 kDa range (**B**); zoomed spectra over the *m*/*z* 2.5–5.0 kDa range (**C**); zoomed spectra over the *m*/*z* 5–7.5 kDa range (**D**); zoomed spectra over the *m*/*z* 7.5–10 kDa range (**E**).

**Figure 3:**
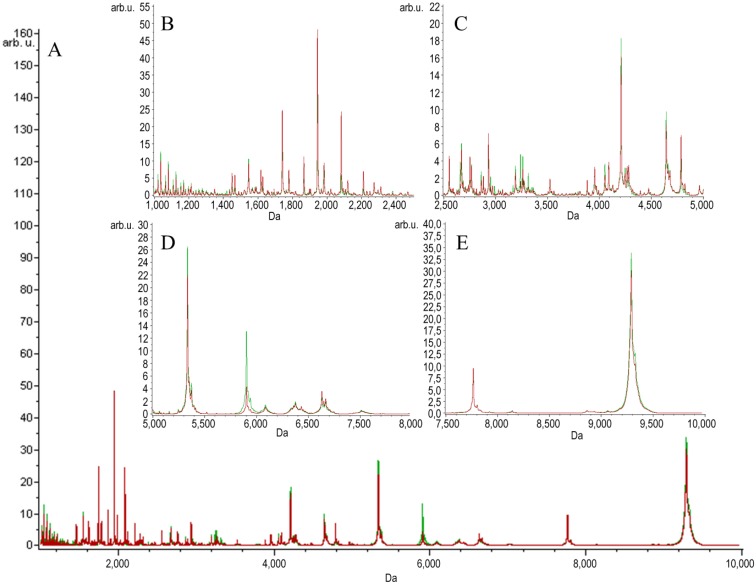
Average MALDI-TOF spectra of serum samples of stung (red) and non-stung (green) individuals pretreated with magnetic beads based on weak-cation exchange (MB-WCX) over the full scan range of *m*/*z* 1–10 kDa (**A**); zoomed spectra over the *m*/*z* 1–2.5 kDa range (**B**); zoomed spectra over the *m*/*z* 2.5–5.0 kDa range (**C**); zoomed spectra over the *m/z* 5–7.5 kDa range (**D**); zoomed spectra over the *m*/*z* 7.5–10 kDa range (**E**).

Two groups of serum samples obtained from 27 beekeepers (24 male, three female) were included in our study. The first group of samples was collected within 3 h after a bee sting (stung beekeepers). The samples were collected from the same person a second time after at least six weeks after the last bee sting, a minimum of six weeks from the end of the beekeeping season (non-stung beekeepers). MALDI-TOF MS combined with Omix, ZipTips and MB-WCX enrichment strategies were used in the study to detect LMW protein/peptide profile spectra in the mass range of 1–10 kDa. The mechanism of the magnetic beads used relies on weak-cation exchange. The MB-WCX kit is based on super-paramagnetic micro-particles, which have negatively-charged functional groups at the surface of the beads [21]. In our study, we used two C18 SPE micropipette tips made by different manufacturers: Omix (Agilent Technologies, Great Britain) and ZipTip (Millipore, NH, USA), which are based on reversed-phase chromatography. The compounds are separated due to their chemical and physical properties, which determine their separation between a mobile liquid phase and a solid stationary phase. The compounds, which are not bound, are washed away. Finally, the bound molecules are eluted from the solid phase by the elution solvent [22,23]. After normalization and alignment of the all processed spectra, with a signal-to-noise threshold equal to or greater than five on the average spectrum, 149 unique peaks were detected in the Omix dataset, 153 unique peaks in the ZipTips dataset and 127 unique peaks in the MB-WCX dataset. MALDI-TOF-MS averaged peptidome profiles obtained using Omix, ZipTips and MB-WCX are shown in Figure 1, Figure 2 and Figure 3, respectively. The samples were classified, and discriminative models were established by using the quick classifier (QC), genetic algorithm (GA) and supervised neural network (SNN) algorithms in ClinProTools software to analyze all of the detected peaks. In order to assess the influence of time series, *in vitro* diagnostic tests for allergy to bee venom were performed in our earlier studies [24]. The diagnostic tests were carried out directly after a bee sting and after at least six weeks after the sting and showed no significant differences in the levels of total IgE antibodies, honeybee venom-specific IgE antibodies, phospholipase A_2_ (the main allergen of honeybee venom)-specific IgE antibodies, serum tryptase and honeybee venom-specific IgG4 antibodies.

To identify the discriminatory power of all detected peaks, the QC algorithm was used. QC is a univariate sorting algorithm. For proper classification of the peak, averages of the peak areas are stored in the model together with *p*-values obtained from a *t*-test. The peak areas are sorted per peak. Then, a weighted average over all peaks is calculated [25]. The best detection value of this model was obtained for the samples pretreated with the Omix strategy (cross-validation: 94.81%; recognition capability: 94.65%). However, for the samples pretreated with ZipTips and MB, applying this algorithm also led to high detection values (Table 1). Using multivariate analysis (GA and SNN) also enabled distinguishing the groups of the stung and non-stung individuals. The genetic algorithm is used to select a combination of peaks that are most relevant for the separation. During the selection of the peaks, the best capable peak combinations are taken into account. This is done by optimizing a function, which aims at optimal class separation with high variance between classes [25,26]. By applying GA for MS data analysis, it was shown that the spectra obtained after sample enrichment using Omix allowed building a model of higher average cross-validation (97.01%) and average recognition capability (100%) compared to the spectra obtained using the magnetic beads and ZipTips strategy (Table 1). SNN is the algorithm based on the classification of the prototype. It identifies some characteristic spectra of each class. Spectra are called prototypes and can be considered as prototypical samples of that class [25]. The SNN algorithm allowed efficiently discriminating stung and non-stung individuals. Comparing all of the enrichment strategies used, the highest cross-validation (97.31%) and recognition capability (100%) was obtained for Omix. 

Feng Qiu *et al.* chose MB-WCX as the best magnetic bead for pre-extraction samples for proteomic analysis in breast cancer research [20]. This method of sample purification is being increasingly used as a relatively simple technique [26]. However, there have been reports criticizing the reproducibility and robustness of magnetic beads [27,28]. Ali Tiss *et al.* proved that the spectra obtained for samples analyzed using ZipTips showed lower background noise and better signal-to-noise ratios compared to the four types of magnetic beads; while the achieved MS profiles are very comparable between samples treated with pipette tips pre-packed with solid phase material, ZipTips and Omix [29], which is in agreement with our findings.

**Table 1:** Statistical analysis of MALDI-TOF spectra of serum samples of stung and non-stung individuals pretreated with Omix, ZipTips and MB-WCX. QC, quick classifier; GA, genetic algorithm; SNN, supervised neural network.

Sample enrichment strategy	Algorithm	Cross validation (%)	Recognition capability (%)	Number of rejected spectra (%)
Omix	QC	94.81	94.65	12.96
	GA	97.01	100.00	
	SNN	97.31	100.00	
ZipTips	QC	84.97	87.20	0.00
	GA	87.08	97.52	
	SNN	53.57	50.57	
MB-WCX	QC	80.41	85.64	0.00
	GA	90.54	99.38	
	SNN	85.16	98.13	

It is noteworthy that the amount of excluded spectra is also a critical element in the evaluation of the different methods used in peptide profiling. The least number of excluded spectra (0%) was obtained for samples analyzed using MB and ZipTips. For samples pretreated with Omix, the average number of rejected spectra was 12.96%. From earlier studies, it is known that the number of failures (shown by the number of rejected spectra) is lower for samples purified with ZipTips compared to MB [29]. ROC curve analysis was used both for the classification and significant feature selection. This diagnostic performance displays the relation between sensitivity and specificity at different thresholds. The area under the curve (AUC) indicates the diagnostic accuracy of the test methods, the greater the area (value close to one), the more precise the method [30]. The ROC curve indicated that using the Omix enrichment strategy allowed obtaining as many as 10 peptides that demonstrated an AUC value of 1.0, which yields the best possible prediction method characterized by 100% sensitivity and 100% specificity. Their *m*/*z* values were: 1277.60 Da, 1436.01 Da, 5656.11 Da, 6432.84 Da, 6472.15 Da, 6528.35 Da, 6631.08 Da, 6432.89 Da, 6631.19 Da and 6632.70 Da. For serum samples pretreated with magnetic beads, the highest discrimination quality (AUC: 0.87) was shown by a peptide with a mass of 2120.29 Da, whereas using ZipTips allowed reaching an AUC of 0.9 for a peptide mass of 1277.49 Da. The sensitivities and specificities of the discrimination quality of peaks obtained in our study were satisfactory in all sample enrichment strategies used according to the data presented in the available literature [20,31,32].

Every single peptide indicator has an inherent specificity and sensitivity that cannot be improved. However, multiple indicators can be combined to achieve improvement in research parameters. In our study, different diagnostic models generated by the QC, GA and SNN algorithms analysis comprised at least several indicators. The *m*/*z* values of discriminating peaks from three models (QC, GA, SNN) are shown in Table 2. Analyzing samples pretreated with Omix, it was shown that there is one peak with an *m*/*z* value of 1299.62 Da, which is by all three models. Applying MB as the enrichment strategy also allowed obtaining one mutual discriminating peak by all three models with an *m*/*z* value of 3240.99 Da. In the analysis using each of the depletion methods, there are several mutual peaks between at least two models. The various *m/z* values of discriminating peaks are caused by different peak classification in the algorithms used. Applying these established models, serum samples derived from stung individuals could be distinguished from non-stung controls.

Since the first publication of Lancet in 2002, SELDI (Surface enhanced laser desorption ionization)has been explored for cancer diagnosis [33]. After this article, several papers have been published that confirmed both the MALDI and SELDI methods to be promising diagnostic tools in different diseases [34,35,36,37]. At the same time, it also suffered from criticism about reproducibility from different labs. However, our findings showed that MALDI-TOF-MS is a useful method for investigating the organism’s response to physiological or pathophysiological stimuli at the proteomic or peptidomic level. All of the statistical algorithms used in this study allow distinguishing the analyzed groups with high statistical significance, which confirms the influence of honeybee sting on the serum peptidome profile.

**Table 2:** Discriminating peaks between stung and non-stung individuals for serum samples pretreated with Omix, ZipTips and MB-WCX.

Enrichment Strategy	Omix			Ziptips			MB-WCX		
Algorithm	QC	GA	SNN	QC	GA	SNN	QC	GA	SNN
*m/z* value of peaks used for classification (Da)	1277.63	1299.62	1656.15	1037.36	1993.78	8933.73	1450.64	4661.58	3240.99
	1299.62	1458.18	1261.58	1078.03	1037.36		1628.00	1779.41	1866.30
	1436.04	1332.67	6432.85	1125.57	2379.82		1779.41	3240.99	1628.00
	1505.18	1217.39	2755.95	1207.37	1299.90		1866.30	3934.87	2883.66
	1656.15	8766.23	1299.62	1277.47	1217.26		2082.49	1153.78	2120.27
	4210.32		1277.63	1299.90			2120.27		2082.49
	4467.44		1436.04	1364.67			2210.55		1450.64
	4568.85		1505.18	1466.78			2554.59		1617.58
	4711.11		7157.01	1584.84			2660.80		3955.05
	6432.85		1217.39	1897.73			2754.17		4053.49
	6450.18		2973.00	4466.21			3240.99		2092.96
	6472.33		1904.97	8933.73			4053.49		5001.79
	6528.33		7766.34				5940.75		9062.91
	6631.12		6631.12						2688.19
	6648.76								6509.33
	6670.30								6527.47
	7672.67								8863.22
	8919.94								4963.28
	9136.06								
	9290.45								
	9310.43								
	9333.33								
	9384.58								
	9425.00								

## 3. Experimental Section

### 3.1. Study Participants and Serum Samples

In the study, the participating volunteers were recruited at the beekeepers’ meetings. Two groups of serum samples obtained from 27 beekeepers (24 male, 3 female) were included in our study. The first group of samples was collected within 3 h after a bee sting (stung beekeepers). The samples were collected from the same person a second time after at least 6 weeks after the last bee sting, a minimum of 6 weeks from the end of the beekeeping season (non-stung beekeepers). The blood sampling was carried out after overnight fasting to reduce the influence of food components on the peptide profile. The volunteers’ age range was from 20–80 years. The storage temperature of the samples was −80 °C until the analysis. The study was conducted with the approval of the Bioethics Committee of the Poznan University of Medical Sciences, Poland (Resolution No. 324/11) and fulfilled the requirements of the Helsinki declaration. Consent to participate in the study was written by all beekeepers.

### 3.2. Chemicals and Reagents

Trifluoroacetic acid (TFA), ultrapure water and α-cyano-4-hydroxycinnamic acid (HCCA) were supplied by Sigma Aldrich (St. Louis, MO, USA). Ethanol, isopropanol and acetonitrile (ACN) were supplied by J.T. Baker (Center Valley, PA, USA). All used reagents were of analytical grade or better.

### 3.3. MALDI-TOF-MS Analysis

Directly before MALDI-TOF-MS analysis, large molecular weight proteins were removed from serum samples. Depletion was performed using micropipette tips C18 Omix (Agilent Technologies, Waldbronn, Germany) [22], ZipTip (Millipore, Bedford, MA, USA) [23] and magnetic beads WCX (Bruker, Bremen, Germany) [21], according to the manufacturer’s instruction. Then, samples were mixed with 0.5 µL of one of the matrix solutions, α-cyano-4-hyroxycinnamic acid (HCCA), and put directly onto the MALDI plate (AnchorChip, Bruker Daltonics, Bremen, Germany). Each sample was spotted in triplicate on the plate. Samples were analyzed using an UltrafleXtreme MALDI-TOF mass spectrometer (Bruker Daltonics, Bremen, Germay). The analyzer worked in the linear mode, and positive ions were recorded in a mass range of *m/z* between 1 kDa and 10 kDa. Typical instrument settings were: Ion Source 1, 25.09 kV; Ion Source 2, 23.80 kV; lens, 6.40 kV; pulsed ion extraction, 260 ns; matrix suppression mass cut off, *m*/*z* 700 Da for a mass range 1–10 kDa. Two thousand spectra (laser shots) were picked up for one analysis. For external calibration of the mass spectrometer, the ClinProt standards (1:5 mixture *v*/*v* of Peptide Calibration Standard and Protein Calibration Standard I) were analyzed. The average mass deviation was better than 100 ppm. Each sample was analyzed three times. Inter-day and intra-day reproducibility of MS results on three representative serum samples have been analyzed in triplicate in three consecutive days and three times within a day. The data have shown that the coefficient of variance (CV) for five selected *m/z* peaks with the highest amplitude was less than 10%. The data collection was obtained with FlexControl 3.4 software (Bruker Daltonics, Bremen, Germany, 2011), and the spectra were saved automatically in FlexAnalysis 3.4 software (Bruker Daltonics, Bremen Germany, 2011).

### 3.4. Statistical Analysis

In order to determine the optimal model allowing the discrimination of the analyzed samples, chemometric software for biomarker detection ClinProTools 3.0 (Bruker Daltonics, Bremen, Germany, 2011) was used. The baseline was set by the “Top Hat Baseline” algorithm. Smoothing of the spectra was obtained by Savitzky–Golay. For the samples’ classification, the following algorithms were used: GA, QC and SNN. For these algorithms’ cross-validation, recognition capability and the number of rejected spectra were calculated. These indicators of the model’s performance are useful predictors of the model’s capability to distinguish between two studied groups. The cross-validation values reflect the model’s ability to handle variability among test spectra. It can be used to predict how the model will behave in the future. In our research, random cross-validation was set. A random subset of data points is taken over all classes and left out of the model generation procedure. For model generation, the remaining points are used. The absent data points are classified against the model. The obtained classification results are stored for the model. This formula is repeated for a defined number of iterations. The following settings have been used: random mode, 20% to leave out, 10 iterations. The results are averaged and returned as the prediction capability. The specificity and sensitivity of a test and evaluation of the discrimination quality of the peak were calculated by the statistical method receiver operating characteristic (ROC) curve. The generic principle behind this diagnostic performance is to give a graphical overview between sensitivity and specificity at different thresholds. The best prediction method would obtain 100% sensitivity and 100% specificity. The precise way to characterize the ROC curve is the area under the curve (AUC). It indicates the diagnostic accuracy of the test methods: the greater the area (value close to 1), the more precise the method. In ClinProTools, the ROC curve is used as a test separating two groups. The threshold is represented by the peak area or the intensity of the peak. On the diagram on the x-axis, the specificity is given (false positives) and on the y-axis the sensitivity (true positives). Statistical significance was assumed when the *p*-value was <0.001.

## 4. Conclusions

It can be concluded that the implementation of different sample enrichment strategies (Omix, ZipTips and MB-WCX) linked with MALDI TOF MS and different chemometric algorithms allowed obtaining high differentiation between stung and non-stung individuals. Furthermore, it has been shown that *Hymenoptera* sting changes the serum peptidomic profile. Mass spectrometry-based serum peptidomic profiling is a technique that may broaden the understanding of the human body response to honeybee venom. Due to the fact that our pilot study was carried out on relatively small datasets, it is necessary to conduct further proteomic research of the response to honeybee sting on a larger group of samples.
